# Welfare standards in hospital mergers

**DOI:** 10.1007/s10198-012-0403-x

**Published:** 2012-06-12

**Authors:** Katalin Katona, Marcel Canoy

**Affiliations:** 1 Dutch Healthcare Authority, PO Box 3017, 3502 GA Utrecht, The Netherlands; 2 TILEC, Tilburg University, PO Box 90153, 5000 LE Tilburg, The Netherlands; 3 Ecorys, Watermanweg 44, 3067 GG Rotterdam, The Netherlands

**Keywords:** Merger control, Hospital merger, Welfare standard, Externality, I11, I18, L44

## Abstract

There is a broad literature on the consequences of applying different welfare standards in merger control. Total welfare is usually defined as the sum of consumer and provider surplus, i.e., potential external effects are not considered. The general result is then that consumer welfare is a more restrictive standard than total welfare, which is advantageous in certain situations. This relationship between the two standards is not necessarily true when the merger has significant external effects. We model mergers on hospital markets and allow for not-profit-maximizing behavior of providers and mandatory health insurance. Mandatory health insurance detaches the financial and consumption side of health care markets, and the concept consumer in merger control becomes non-evident. Patients not visiting the merging hospitals still are affected by price changes through their insurance premiums. External financial effects emerge on not directly affected consumers. We show that applying a restricted interpretation of consumer (neglecting externality) in health care merger control can reverse the relation between the two standards; consumer welfare standard can be weaker than total welfare. Consequently, applying the wrong standard can lead to both clearing socially undesirable and to blocking socially desirable mergers. The possible negative consequences of applying a simple consumer welfare standard in merger control can be even stronger when hospitals maximize quality and put less weight on financial considerations. We also investigate the implications of these results for the practice of merger control.

## Introduction

Competition authorities that need to decide on mergers have some leeway as to the criteria for their assessment of welfare. Most authorities have made a choice for consumer welfare. However, there are some countries, such as Canada and Australia, where merger control seems to follow the principles of total welfare [1]. There is a long standing discussion in the economic literature on whether one or the other standard is preferable. The question is addressed both from practical and theoretical perspectives. We contribute to this discussion by pointing out that specific features of the health care sector, making externalities emerge, reverse some key results.

In the simplest case, total welfare in an economy is calculated as the sum of consumer surplus and producer surplus [2]. In some industries, however, other components of welfare such as external effects on not directly affected consumers may arise as significant. We could take examples from environmental economics (assuming that the merger influences the magnitude of the externality, e.g., pollution), from the financial sector (thinking of the effect of a merger on the stability of the whole system) or, as we do in this article, from the health care (and insurance) sector. Facing a merger case with significant external effects, competition authorities should not lean on general results but should take into account the peculiarities of the market. As shown in this article, neglecting the external effects can alter the conclusion on the preference of consumer or total welfare standard in merger control.

The question of whether to explicitly include externalities in merger analysis of health care markets raises another ambiguity. The relationship between health services and health insurance markets makes non-evident who the consumers are in a merger of providers (e.g., hospitals). The exact definition of the consumer determines whether externalities are implicitly taken into account or their explicit consideration is needed in the analysis. Besides presenting the consequences of neglecting to include important externalities in the analysis, we also embrace the topic of how potential non-profit-maximizing behavior of hospitals affects the results.

Merger control of hospitals is a relatively new for competition authorities, and is less analyzed in the literature on optimal welfare standards. Hospital markets are liberalized in a number of countries (e.g., Switzerland, Netherlands, US) making it a sector that falls under competition law scrutiny. 1

Hospital markets have certain specific features that require attention. First, hospitals are not necessarily striving for maximum profits. When providers attach great weight to quality arguments in their merger decisions 2, it is more probable that mergers of socially undesirable (i.e., excessive) quality improvements are initiated. Second, the market (in many countries) is characterized by the fact that patients are insured. The concept ‘consumer’ becomes non-evident when insurance is mandatory, premiums are uniform regardless of heterogeneity among consumers, and in the absence of significant co-payments. Some of these elements, i.e., mandatory insurance, uniform premiums and absence of significant co-payments, apply to other (non-health) insurance markets as well. However, the combination of all the three characteristics particularly features health care insurance (e.g., in Germany, France and The Netherlands).

Patients receive treatment from providers but do not pay directly for services. Insurers reimburse providers and collect premiums from their clients. The level of the premium is, however, independent from individual consumption 3, thus detaching the financial and consumption side of the market for hospital services.

Taking a hospital’s perspective, a hospital has, on the one hand, clients to whom it offers services (patients) and on the other hand clients from whom it receives reimbursement for the same services (insurers). The group of consumers receiving the services and the group paying for it through health insurance typically do not coincide. This level of detachment of monetary transactions and service flow makes the correct definition of the consumer non-evident. Theoretically, there could be many different definitions of a ’consumer’. Moving from a narrowly to a broadly defined group, a consumer can be: one that actually visits the given hospital; one that might visit it; or one that, through the insurance premium, pays for the services offered by the given hospital. Subsequent definitions broaden the group of consumers and consider the financial effects in increasing levels. Since insurance spreads health care expenditure across all its clients, only the most extended definition allows one to consider the whole financial effect of a merger. If we use a narrower definition, we disregard the external effects of uniform insurance premiums.

Our contribution to the literature is that we explicitly model these specific hospital market features, by which we consider the consequences for the analysis of potential externality and not-profit-maximizing behavior. We define two groups of consumers. First, the broadly defined group ‘indirectly affected consumers’ contains all clients of insurance companies. Some of these consumers are likely to never visit the merged hospital (e.g., because they live far away), and are not affected by possible changes in the service level of the hospital. For example, these patients do not benefit from a quality improvement or from the introduction of a new technology in the merged hospital. They are, however, affected indirectly through the financial effects. Since they purchase health insurance, all these consumers share the burden of a possible rise in insurance premium; they are exposed to an external effect of the merger. (The previously mentioned group of consumers who at the moment are not sick but in the future might visit the hospital are also included in this category. They draw some benefits from the quality improvement—we will define this as option value in the section on Application in merger control—because in case of illness they can expect better treatment. They are also affected by potential price increases through their health insurance.) Second, the narrowly defined group ‘directly affected consumers’, which is also part of the previous broader group, contains only the clients of insurance companies that actually visit the merged hospital. These consumers are not only exposed to financial effects, but are also affected directly by possible changes in quality. However, when using this narrow definition, we do not consider the whole financial effect of the merger. Specifically, the premium paid by consumers who do not visit the merged hospital is not considered. A part of the financial effect appears as an externality in the analysis.

Depending on the definition of ‘consumer’, the exact meaning of consumer welfare standard also changes. Applying a narrow definition, i.e., neglecting the externality, the consumer welfare (CW) standard can result in a more lenient criterion than the total welfare (TW) standard, which is in contrast to the general view in the literature. In markets where consumers obtain the benefits and pay the costs of their consumption, the CW standard has been equal to or tougher than the TW standard. Many theoretical models (e.g., [4, 5]) have built on this feature by showing the superiority of the CW standard in a number of circumstances. We show that the externality effects on health care markets stemming from insurance can reverse the relationship between CW and TW standards, which questions the generality of literature claims.

From a policy perspective, a narrow definition of the ‘consumer’ in the CW standard can lead to both approving socially undesirable mergers as well as blocking socially desirable mergers. A CW standard, which applies the most extended definition of ‘consumer’ and so implicitly includes the external effects, repairs this problem. However, it requires one to consider effects that are potentially external to the relevant hospital market of the merger. In the process of merger control, this dilemma appears in the evaluation of the potential positive (quality) effects of a merger against the negative (price) effects. The externality caused by insurance is reflected in the diverging valuation of quality improvements by different groups of consumers. The method used to value potential quality improvements and aggregate it across all consumers influences the effectiveness of the merger standard.

The next subsection reviews the economic literature that investigates the reason for different welfare standards in merger control. Related literature describes the model. The following section, Model, summarizes the results of our model and discusses the consequences of applying different merger standards from a theoretical perspective. Policy implications and relevance to the current practices are described in Application in merger control, followed by Conclusions.

### Related literature

There are two branches of economic literature analyzing which welfare standard is optimal in competition policy analysis. One compares practical and direct effects of applying TW or CW while the other branch applies the agency framework to analyze the decision problem of competition authorities. We summarize the arguments of both approaches. At the end of this section, we relate the results of the literature on non-for-profit hospital mergers to our findings.

Articles on the practical approach (e.g., [6–8]) focus broadly on two aspects of a merger: changes in efficiency of production and (re)distribution among different groups of society such as providers and consumers. Regarding efficiency, these studies analyze whether a merger offers opportunities to produce more quantity or better quality given the scarce resources in the economy. The TW and CW standards differ in what exactly are considered as efficiency gains. While the TW standard values every efficiency improvement, the CW standard acknowledges only gains that are passed on to consumers. Fixed cost savings, for example, may outweigh the anticompetitive effects of a merger according to the TW standard but do not contribute to the CW standard. The CW standard ignores some efficiency gains and gives priority to distributional aspects. Using the CW standard can lead to a situation where consumers are the final beneficiaries of the merger, but it forgoes some efficiencies that would benefit solely providers. The TW standard does not consider (re)distributional effects but evaluates mergers solely on efficiency considerations. There is no clear conclusion in the literature as to which approach is in line with the goals of competition policy.

Articles in the other branch of the literature look at the merger control process as a whole and apply principal-agent theory (e.g., [4, 5, 9, 10]). They assume that the ultimate goal of society is to maximize TW. In these models, the competition authority is an agent that controls mergers according to a given welfare standard. The focus of these analyses lies in the consequence of choosing TW or CW standard as the objective function of the competition authority. Despite the fact that the final goal is to maximize total welfare, it can sometimes be achieved by defining CW as the objective function for the agent authority. This can be explained by a general result according to which CW is a more restrictive standard than TW. Besanko and Spulber [4] and Lyons [5] both build on this characteristic of CW, and show under which conditions the CW standard achieves higher TW than the TW standard.

Besanko and Spulber [4] apply a model of asymmetric information to show that a tougher merger standard than the TW standard increases the societal gain from a merger. Since authorities cannot estimate perfectly the welfare consequences of a merger, their decision is a random variable from the firms’ perspective. Furthermore, rejection of a merger has a higher probability when the CW standard (a tougher criterion) is applied. Because preparing and submitting a merger proposal is costly, firms initiate mergers that they expect to be accepted. In the case of a tougher merger standard, this results in self-selection toward socially preferable merger alternatives. The key elements in this model leading to the preference of CW standard are the costly procedure and asymmetric information.

Lyons [5] derives the relative advantage of one or the other welfare standard from the diverging treatment of changes in fixed costs. CW standard incorporates cost reductions only if they are passed on to consumers. Therefore, fixed cost reductions are excluded from the CW analysis. Welfare gains from mergers in Lyons’ article are described as the ratio of anticompetitive effects and fixed cost reductions. The CW standard is a tougher standard because potential anticompetitive effects cannot be compensated by cost reductions. Since firms prefer mergers with anticompetitive effects (higher prices), the CW standard is more likely to reject the first proposal of firms than the TW standard. The desirability of rejection in the long term depends on alternative mergers. If the subsequent proposal of firms yields a higher TW, then rejection was a desirable decision. If the alternative is a socially less beneficial merger, then approval by TW standard is a better strategy. Lyons [5] models a given industry structure and analyses sequential mergers to find the equilibrium structure conditional on the merger standard.

These articles have considered general sectors without significant externalities, a set-up that does not fit health care markets. Calem et al. [11] focus on distinguishing different welfare measures specific to health care markets. They emphasize two distinguishing characteristics of hospital (and general health care) markets. First, health care insurance causes moral hazard in the consumption of hospital services to the extent of the co-payment rate. Second, hospitals may be non-profit; specifically, they may maximize output instead of profit. Considering these characteristics of hospital markets, they compare the effects of a merger on consumer surplus (gain from hospital services minus co-payments paid by consumers), net social surplus (gain from hospital services minus price paid by the insurer) and gross social surplus (gains from hospital services minus costs born by hospitals). They model quality competition among hospitals, which may yield over-production of quality because of moral hazard or the non-profit nature of hospitals. Consequently, a merger may be gross social welfare enhancing since it reduces quality competition and restricts excess quality. Considering only consumer surplus, which reduces with decreasing quality, can be misleading when evaluating hospital mergers. These results are health care specific but are not linked explicitly to the literature on merger control. Our article makes this last step too. Similar to Calem et al. [11], we compare various welfare concepts applied to health care markets. In addition, we look explicitly at the consequences of using these measures in merger control.

Finally, we discuss the strand of literature that investigates whether not-for-profit (NFP) organizations should be treated differently in merger control than for-profits (FP)organizations. Several articles show that the behavior of NFP firms can be interpreted as a profit-maximizing behavior with lower perceived costs. Beside monetary profit, NFP firms gain additional utility from production, which makes them accept higher costs for the same level of production. This attribute of NFP organizations appears in our model as well. Both theoretical (e.g., [12, 13]) and empirical (e.g., [14, 15]) articles conclude that NFP hospitals exploit their market power in a similar way as their FP counterparts.

Prüfer [16], however, shows that this result depends on the assumptions made on the maximand of the NFP firms. In his article, NFP firms with an owner preferring high quality produce excessive quality (from the societal perspective). The merger (to monopoly) eliminates competition, which indirectly makes the firm produce lower quality, in this way increasing the total welfare. Prüfer [16] draws attention to the importance of examining the objective of the owners of merging NFP firms when assessing the effects on society.

It is important to note that the NFP status of hospitals differs from possible not-profit-maximizing behavior. NFP status is a definition used in the context of taxation and refers roughly to two rules. NFP hospitals enjoy exemption from taxation, and they are not allowed to pay rents to their owners (non-distribution constraint). In contrast, not-profit-maximizing behavior refers to the objective function of the hospital that is revealed in its decisions. In this article, we consider this second possibility but do not discuss the case of NFP status. Similar to the literature above, our model shows that merged quality-maximizing hospitals exploit their market power just as their profit-maximizing counterparts but their decisions on merger can differ significantly.

## Model

Our static model includes three players; hospitals deciding whether to initiate a merger, consumers paying the insurance premium and choosing a hospital when they fall ill, and a competition authority blocking or approving the merger. The standard that the competition authority applies in merger control is either the TW standard or a version of the CW standard. In both cases, the authority approves the merger if the standard indicates net gains and blocks the merger if the standard indicates net losses. We do not model the insurance market explicitly. This assumption is not restrictive for the purpose of this study.

### Hospital market and consumer preferences

We characterize the market for hospital services and consumer preferences by applying the circular city model. Distance to a hospital is an important choice factor of consumers (see, e.g., [17]), which makes substitutability of providers asymmetric, i.e., dependent on distance from the consumer’s location. Location models fit this characteristic of the market. Furthermore, we focus on effects of a merger and consider hospitals in any other aspect symmetric. The circular model, in contrast to the linear model, allows for this. 4

Let the *n* hospitals offering treatment to patients be located on a circle of unit circumference at equal distance from each other. Besides horizontal differentiation, hospitals may also vary in quality of services offered. Patients 5 are distributed uniformly on the circumference of the circle. We assume that every patient prefers hospital services of higher quality to that of lower quality and that they dislike travelling. They trade-off quality and distance from the hospital uniformly in ratio *t*. Patients do not pay directly for their treatment; therefore, price does not play a role in their hospital choice. Thus, the utility derived from receiving hospital treatment includes two terms; the quality of the services in the visited hospital (*q*_i_), and distance to the hospital (*x*).

1 $$ U=q_i-tx $$

The demand for hospital *i*’s services (Eq. 2) consists of the sum of two ‘half demands’: the demand in the market segments where hospital *i* competes with hospital *i* + 1 and *i* − 1 respectively. In each segment, the demand is derived by determining the position of the indifferent patient based on the utility function (Eq. 1).

2 $$ D_i(q_i,q_{i-1},q_{i+1},t)=\frac{1}{n}+\frac{q_i-q_{i-1}}{2t} +\frac{q_i-q_{i+1}}{2t} $$

Equation (3) describes the utility that patients of hospital *i* derive from their visit. We refer to this value as patient welfare produced by a given hospital (PW_*i*_). Equation (4) defines the (total) patient welfare (PW), which is the sum of the welfare produced by each hospital.

3 $$ {\rm PW}_i=\int\limits_0^{\frac{1}{2n}+\frac{q_i-q_{i-1}}{2t}} \! (q_i-tx) {\rm d}x + \int\limits_0^{\frac{1}{2n}+\frac{q_i-q_{i+1}}{2t}} \! (q_i-tx)\, {\rm d}x $$

4 $$ {\rm PW}=\sum_{i=1}^{n} {\rm PW}_{i} $$

### Insurance market and hospital-insurer bargaining

We do not model the insurance market and the hospital-insurer relationship explicitly but make some simplifying assumptions. In the insurance market, we assume Bertrand competition among symmetric firms, which results in premiums at the level of the uniform marginal cost. The single role of the insurance market in this model is to pool patients’ health care expenditure and set a uniform premium for all consumers. In this model, prices between insurers and hospitals are assumed to be the result of negotiation. Instead of modeling the negotiations explicitly, we make two assumptions on the outcomes and incorporate these simplified solutions in the further steps of the model. These assumptions are common in the literature and are not restrictive for this model.

First, negotiated prices between hospitals and insurers consists of two parts: reimbursement of the costs of the hospital and a share in the net gain from concluding the contract 6. The net gain is here defined similarly to Capps et al. [18], i.e., it equals the added value that the given hospital brings to the insurer’s network minus the additional expenditure (or saving) caused by including the given hospital in the network. Including an extra, less efficient, hospital to the insurer’s network yields added value to patients because they need to travel less. At the same time, including a less efficient hospital means extra costs for the insurer. Therefore, when concluding one more contract, the insurer’s clients have to travel less but are cured more expensively. The difference of these two effects is the net gain from concluding the contract. Negotiating hospital-insurer pairs bargain about the division of this net gain.

Formally, the added value of a given hospital is defined as the total patient welfare assuming patients may attend all hospitals in the market minus the total patient welfare assuming that patients may attend any but the given hospital in the market. This value represents the added utility that patients derive from the existence of that given hospital. Note that this formula yields higher added value for a hospital of high quality or in an isolated location than for a hospital of average quality in a densely populated location. The underlying intuition is that dropping the high-quality low-density hospital from the market leads to consumers substituting it for a hospital of considerably lower quality or for one lying relatively far away. Such substitution means loss of utility for consumers. Additional expenditure is defined similarly; costs of the insurer when the hospital is included in its network minus the costs assuming that the hospital is not part of the network.

Second, in order to keep the model simple, we assume that the insurer and the hospital share the net gain from concluding the contract in a given proportion, namely 50–50 7. We apply the following formula to determine the price of hospital *i*’s service (*w*_*i*_)

$$ w_i=c_i+\left(\frac{{\rm PW- PW}_{-i}}{D_i}-\frac{{\rm TE- TE}_{-i}}{D_i} \right) \frac{1}{2} $$

where *c*_i_ denotes the constant average cost of hospital *i*, TE denotes the total expenditures of the insurer, which is defined as TE = ∑_*i*=1_^*n*^ *D*_i_ *w*_i_. PW_−i_ denotes the total PW when hospital *i* is not in the market and similarly TE_−i_ is the total expenditure of the insurer when hospital *i* is not in the market. 8 Note that we assume hospitals to agree on the same price with all insurers, i.e., a hospital has a single price. Since insurers are symmetric in the model, this is a logical assumption.

When modeling a merger between hospitals, we apply the notion of a merger as defined in the property rights literature (e.g., [19]). The idea is that integration implies the shift of ownership rights between the merging entities. The hospital that is taken over no longer acts as an individual entity, and the new owner of the merged hospital has disposal of both hospital locations. Therefore, a single decision maker negotiates with the insurer following the merger. If they fail to agree, both hospital locations become unavailable for patients. PW_−i_ is thus calculated by dropping both hospitals from the network. Therefore, patients have to travel further for a substitute than before merger, and the added value of the hospital increases. A merger leads to higher prices, ceteris paribus, reflecting the increased market power of the merged hospital.

Note that the price depends only on the value that hospitals add to patient welfare both in the integrated and the non-integrated situation. Hospitals of higher than average quality produce more added value; therefore, they have higher prices. This is, however, independent of the hospital’s objective function 9. Similarly, the price of the merged hospital increases regardless of its maximand. This result is in line with the literature on NFP hospitals, which shows that NFP hospitals exploit their market power similarly to their FP counterparts. Our results coincide with this, although we model a bargaining outcome in contrast to the usually assumed price setting behavior.

### Welfare measures

Based on the exact definition of the ‘consumer’ used in the merger analysis, we identify two different consumer welfare standards. We define ‘simple CW’ as the difference between PW and the share patients pay from health care expenditures. Simple CW measures the direct effect of a merger, and does not consider external effects introduced by health insurance. The concept of the ‘consumer’ is defined here as patients visiting the hospital, which is a narrow definition because it excludes a large group of healthy consumers or consumers in other hospital markets.

The extended definition of ‘consumer’ that we use involves everyone affected by the merger (including the previously defined smaller group), which implies all people covered by the same insurance because, through the uniform premium they pay, they are affected by changes in the hospital prices. Furthermore, we assume that consumers from other hospital markets can also be pooled by the same insurance and so can be affected by the merger. Expenditures can be shared among consumers in a larger region than the hospital market each patient considers. We term the welfare measure calculated as PW derived from hospital services minus total health care expenditure ‘extended CW’.

The difference between simple and extended CW is in the cost component, i.e., the implicit in- or exclusion of external effects. Patients visiting the hospital enjoy all benefits of a potential quality increase (reflected in increasing PW), but pay only a proportion of potential extra costs 10. When considering all consumers affected by the merger, PW is still considered, and costs are also fully taken into account. Calculating the extended CW, we internalize the external effects of insurance on consumers paying premium but not visiting the given hospital.

To formalize the concept of simple CW and extended CW, let us define the ratio

$$ S=\frac{\hbox{Number \, of \, patients \, directly \, affected}}{\hbox{Number of all affected consumers}} $$

Equation (5) defines simple CW, while Eq (6) defines extended CW. Note, that *S* can also be interpreted as the ratio of consumers that are included in the merger analysis. In this way, *S* is a continuous variable that determines the level of externality and the distortion introduced by partial analysis that excludes a group of consumers paying premiums. Equation (6) shows that the extended CW can be written as the sum of simple CW and the externality effect caused by health insurance.

5 $$ {\rm CW}_{\rm simple} = {\rm PW} - S \sum_{i=1}^n D_i w_i $$

6 $$ {\rm CW}_{\rm ext} = {\rm PW} - \sum_{i=1}^n D_i w_i = {\rm PW} - S \sum_{i=1}^n D_i w_i - (1-S) \sum_{i=1}^n D_i w_i $$

TW is defined as the difference of PW and the cost of its production, which equals the sum of the welfare of all groups in the society. 11 Similar to the theoretical strand of the literature, we use TW as a benchmark.

$$ {\rm TW}= {\rm PW}-\sum_{i=1}^n D_i c_i $$

### Objective function of hospitals

Several articles in the literature test possible assumptions on the objective function of NFP hospitals. The assumption of non-profit maximization is investigated by several empirical articles [21–23]. They confirm that the maximand of NFP hospitals is not the monetary profit. Deneffe and Masson [22] and Horwitz [23] find that NFP hospitals are most likely to maximize output or a mix of monetary profit and output, while Chang and Jacobson [21] conclude that the data is the most consistent with the theory of perquisite maximization. Others (e.g., [24, 25]) find that NFP hospitals simultaneously provide more charity or unprofitable care or higher quality than FP, which can be the result of some kind of welfare maximization. Although there are several hypotheses on the real objective function of NFPs, there is no consensus in the literature. In an overview of the empirical literature, Malani et al. [26] conclude that there is not enough evidence to distinguish among different theories on the NFP objective function.

In our model, we assume that hospitals maximize a combination of monetary profits and quality. 12 In one extreme case of our model, hospitals maximize purely the quality level of their care regardless of monetary profits. The other extreme is the pure profit-maximizing behavior. We will refer to hospitals following exclusively the previous strategy as purely-quality-maximizing hospitals, while to hospitals following the latter strategy as purely-profit-maximizing hospitals.

Quality improvement can be a strategy for both the purely-quality- and purely-profit-maximizing type. In contrast to quality-maximizing hospitals where quality improvements increase the objective function of the hospital directly, quality improvement has only an indirect effect on profit-maximizing hospitals. Quality increases the monetary profit through higher market shares and higher prices. The motive of a profit-maximizing hospital for quality improvement differs essentially from the motive of a purely-quality-maximizing hospital.

Specifically, we assume that hospitals maximize the weighted average of monetary profit ($\Uppi$) and quality (*q*). The objective function of hospital *i* is thus:

7 $$ G_i=\alpha \Uppi_i +(1-\alpha )q_i = \alpha D_i(q,t)(w_i-c_i)+(1-\alpha )q_i $$

where $\Uppi_i$ denotes the monetary profit of the hospital, which is demand multiplied by price minus cost. We denote the relative weight of profit maximization to quality maximization in the decisions of the hospital by α. In case of a purely-profit-maximizing hospital, α = 1, while in case of a purely-quality-maximizing hospital, α = 0. Hospitals may aim at both objectives, i.e., they have an eye on costs, but also increase quality purely for its intrinsic value (and not to seek higher market share). We assume that the value of α is the same for all hospitals in the market.

Our definition of the objective function is similar to the model of profit-deviating firms used in Lakdawalla and Philipson [27], although they assume ‘output-preferring’ hospitals. It is important that, similar to the model mentioned above, we do not model the NFP status of hospitals (i.e., the nondistribution constraint), but focus on the profit-deviation behavior. Budget constraint for hospitals such as a constraint for positive monetary profit is not assumed. Lakdawalla and Philipson [27] argues that donors who gain utility from the profit-deviation can cover the negative profits. For the ease of presentation, we also disregard a budget constraint, the inclusion of which would not change our qualitative results.

### Merger decision of hospitals

Two hospitals will initiate a merger only if it results in an increase in their objective function, i.e., their joint gain (*G*_*i*+*j*_) is higher after the merger than before it.

8 $$ \begin{aligned} G_{i+j}^{\rm before} &< G_{i+j}^{\rm after}\\ &\alpha (D_i(w_i-c_i)+D_j(w_j-c_j))+(1-\alpha)\frac{q_{i}+q_{j}}{2}\\ &< \alpha D_{i+j}(w_{i+j}-c_{i+j}) +(1-\alpha)q_{i+j} \end{aligned} $$

Where *q*_*i*+*j*_ is the quality level after merger, *c*_*i*+*j*_ is the cost after the merger, *D*_*i*+*j*_ is the demand of the merged hospital, and *w*_*i*+*j*_ is the price of hospital services calculated for the merged hospital.

As described in Eq. (7), α gives relative weights to monetary and non-monetary benefits in the objective function of hospitals. From Inequality (8) can be seen that a purely-quality-maximizing hospital will initiate any merger with $\frac{q_{i}+q_{j}}{2}<q_{i+j}$ irrespective to costs. Other hospitals (0 < α ≤ 1) consider both quality and cost consequences of a merger. If there are several possible mergers, the hospital chooses the one that ensures the highest gain.

Specifically, we assume two effects of a merger: (1) a merger-specific change in quality, $\Updelta q,$ and (2) a merger-specific change in marginal cost, $\Updelta c$.^13^ The change in quality can be an increase, assuming that, e.g., larger hospitals have a better reputation and so attract better personnel. It can also be a decrease in quality, assuming that cultural differences in the two institutions lower quality in the short run. Costs may decrease or increase owing to a merger. One reason for a decrease could be the scale economy of certain activities. A cost increase, however, is possible, for example, due to higher organizational costs after the merger.

We assume that quality differences after the merger are not so large that it is worthwhile for patients to bypass the nearest hospital (that is to travel more than 1/n of the circle): $\frac{t}{n}>|\Updelta q|$. Furthermore, we consider only mergers between two neighboring hospitals, and assume that the merged hospital does not close any of its locations. The only strategic action on the part of the hospital that we consider explicitly is the decision on the merger: whether and with which (neighboring) hospital to merge.

## Results

To keep the presentation of results simple, we set the initial values of quality and cost level uniform for all hospitals in the market. In this way, we have two quality and two cost levels in the model: *q* and *c* for all hospitals before the merger, which changes to $q+\Updelta q$ and $c+\Updelta c$ for the merged hospital after the merger (but remains *q* and *c* for all other hospitals). Furthermore, we set the transformation rate between quality and traveling at 1 (*t* = 1). Finally, we fix the number of hospitals in the market before the merger at 4 (*n* = 4).

The following equation produces the gain (*G*_*i*+*j*_) that merging hospitals can obtain:

$$ \Updelta G_{i+j}= \alpha\left[\left(\Updelta q-\Updelta c\right) \left(\frac{1}{4}+\frac{\Updelta q}{2} \right)-\frac{(\Updelta q)^2}{4}+\frac{1}{32}\right]+(1-\alpha)\Updelta q $$

As can be seen, even in the absence of cost and quality effects, hospitals gain $\frac{\alpha}{32}$ because of their increased bargaining power. Increasing costs lower the gains from the merger or do not influence it in case of purely-quality-maximizing hospitals. Effects of quality improvements depend on the level of $\Updelta c$ and α. Purely-quality-maximizing hospitals (α = 0) always gain from quality improvements. Hospitals that (partially) maximize monetary profit can gain as well since better quality attracts more patients. More patients generate monetary profit if the hospital has a positive price cost margin. For α = 1, for example, the condition for increase in gains is $\Updelta c< \frac{1}{2}+\Updelta q$.

### Effects on different welfare measures

We compared the effects of a merger for alternative welfare measures: simple and extended CW, and TW. Each of these measures can be used in merger control. However, they yield different conclusions. Simple CW is specific to health care markets. Extended CW corresponds to CW in non-health care markets, and the definition of TW does not differ either. Reasons for and effects of applying CW or TW standard in non-health care markets have been analyzed extensively in the economic literature, while Calem et al. [11] have described some health care specific welfare measures. We relate our findings to these previous results.

Change in (simple) consumer welfare is given by the following equation:

$$ \Updelta {\rm CW}=\Updelta q \left(\frac{1}{2}+\Updelta q \right)-\frac{(\Updelta q)^2}{2}-\frac{S}{2}\left[\Updelta c +\left(\Updelta q-\Updelta c \right) \left(\frac{1}{4}+\Updelta q \right)-\frac{(\Updelta q)^2}{2}+\frac{1}{32}\right] $$

The first term represents the quality gain due to merger, while the last term shows the price effects. The term between ($\frac{(\Updelta q)^2}{2}$) is the loss in travel time; consumers travel more because they sometimes opt for a hospital of better quality that is further away than the nearest hospital. This loss from traveling is always compensated by the gain from higher quality (in the first term) otherwise patients would not choose the more distant hospital.

The last term indicates that *S* mitigates the costs effects, i.e., simple CW is a harder constraint than TW when cost effects are advantageous, and a weaker constraint when price effects are disadvantageous. Simple CW reaches its extreme value in *S* when the number of consumers affected directly and indirectly coincides (*S* = 1). Consumers cover all the health care expenditures, which also yields that simple CW equals the extended CW. Generally, extended CW is the boundary of simple CW since it considers complete cost effects instead of partial analyses.

The concept of ‘consumer welfare’ and ‘net social welfare’ in Calem et al. [11] is similar to our simple and extended CW, respectively. They diverge, however, in modeling the insurance market since Calem et al. [11] do not include insurance premiums in consumer surplus, but consider the out-of-pocket co-payments of consumers. Consequently, their consumer welfare concept captures the cost effects of a merger only to the extent of co-payments. They classify net social welfare as total welfare excluding hospital profits because those are hard to observe or verify. While the calculation of this and our extended CW concept coincides, the underlying idea differs. Our extended CW is not a kind of total welfare, since no profits of providers or insurers are included. If insurers made profit, we would not include it in the calculation. Extended CW is a consumer surplus taking into account all effects of a merger on consumers.

Change in total welfare is given by the following equation:

$$ \Updelta {\rm TW}=\left(\Updelta q - \Updelta c \right)\left(\frac{1}{2}+\Updelta q \right)-\frac{(\Updelta q)^2}{2} $$

The first term represents the net gain from quality and cost effects of a merger while the second term is the loss from further traveling because of quality differences.

Considering non-health care markets, consumer and total welfare changes in the same direction and in the same instances (disregarding some special cases). Exceptions are, for example, changes in fixed costs (not included in consumer welfare) and price discrimination among consumers (total welfare increases while consumer welfare decreases). The reason for the discrepancy between extended CW and TW in this model is the fixed demand (∑_*i*_ *D*_*i*_ = 1), which is specific to health care markets. Because of the insurance market (and the absence of co-payments), patients do not react to price increases, i.e., there is no dead weight loss in the presence of prices above marginal cost. Changes in the price are purely redistributional in terms of gains between hospital sector and consumers; TW remains unchanged while extended CW changes.

The health care specific differences in welfare measures become larger if the group of consumers considered in the analysis is widened.^14^ Patients directly affected by quality changes is the narrowest definition. We expect that they are less concerned with the potential costs of a merger (lowest *S*) because of the high externality effect. Widening the considered group, the potential clients of hospitals, consumers on the hospital market can be included in the analysis. The cost effects of the merger are considered to the extent this group will bear it. External effects due to insurance still can be present if insurers have patients on (and consequently spread health care expenditures across) more hospital markets. A complete consumer welfare analysis would embrace all consumers covered by the same insurance (extended CW). The only welfare effect then excluded is the profit of providers. TW also considers this last aspect of welfare.

### Welfare standards in merger control

Formally, the simple CW standard ignores providers’ profit and the insurance externality. Ignoring providers’ profit makes the standard stronger than the TW standard as can be seen from the results on the extended CW standard. The neglected externality can, however, be negative (when $\Updelta c>0$), which can outweigh the previous effect and make the simple CW standard weaker than the TW standard. In summary, the simple CW standard may both clear undesirable mergers and block desirable ones.

**Fig. 1:**
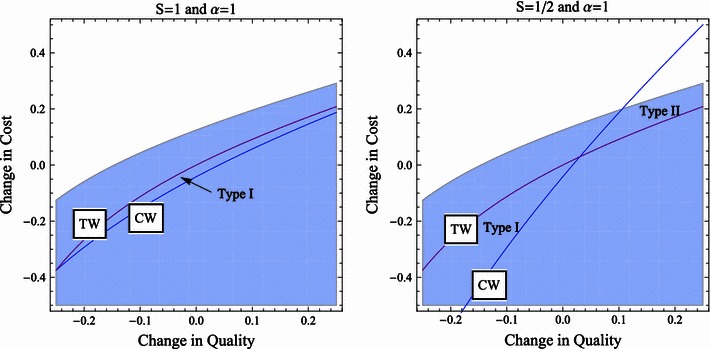
Parameter ranges in which the CW standard commits type I and type II errors, respectively.* Shaded area *Parameter ranges where the merger is profitable for a profit-maximizing hospital (α = 1). We assume no externalities (*S* = 1) in the* left panel* and externalities ($S=\frac{1}{2}$) in the* right panel*

**Fig. 2:**
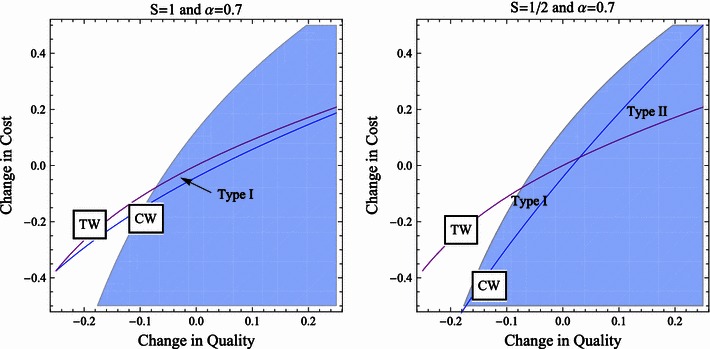
Parameter ranges in which the CW standard commits type I and type II errors, respectively.* Shaded area *Parameter ranges where the merger is profitable for a partially-quality-maximizing hospital (α = 0.7). We assume no externalities (*S* = 1) in the* left panel* and externalities ($S=\frac{1}{2}$) in the* right panel*

While previous literature found reasons why a stronger standard (CW) can be advantageous in merger control, we state that CW can also be too lenient in health care markets, which could lead to clearing undesirable mergers. We describe the intuition of the externality and provide a numerical example illustrated in Figs. 1 and 2. In this way, we disprove that CW standard is always a stronger condition than TW standard. To disprove a claim on generality, a numerical example is sufficient. Notice that the example is by no means an anomaly. We do not provide the general parameter regions where simple CW standard is a weaker standard than TW standard, since that would be technically quite cumbersome and not needed to show the point. Our goal is to show that applying the simple CW standard in health care may have unexpected results.

Figure 1 shows in function of $\Updelta c$ and $\Updelta q$ where a change in TW, CW and merger related hospital gains turns out to be positive. The shaded area depicts merger alternatives that are profitable for purely-profit-maximizing hospitals. This is the set of merger proposals that a competition authority can expect. Below the TW line, the change in total welfare is positive. Applying TW standard, the authority would clear a merger in this parameter range. Applying CW standard, the authority approves every merger alternative below the CW line. On the left panel, CW is calculated as extended CW (*S* = 1), while on the right panel, CW is the simple CW with $S=\frac{1}{2}$. Figure 2 shows similar parameter ranges for partially-quality-maximizing hospitals (α = 0.7).

As seen on the left panel of Fig. 1, the extended CW standard is tougher than the TW standard. Since we have chosen TW as the benchmark, we can say that the extended CW standard commits type I errors: it rejects mergers that would increase TW. Besanko and Spulber [4] build on this characteristic of CW and state that it can contribute to the self-selection of merger proposals that increase total welfare. In the model of Lyons [5], CW is again a tougher standard than TW. That model takes account of alternative mergers and studies the decision of a competition authority from a dynamic perspective. Under the CW standard, the authority is more likely to reject a merger than under the TW standard. This is an advantage when an alternative merger gives higher TW. Thus in both models, the CW standard can commit type I errors, but no type II errors; i.e., it may reject mergers that increase TW, but it does not approve mergers that decrease TW. This idea coincides with our results on the extended CW standard. The simple CW standard, however, may commit type II errors as well, as shown in the right panel of Fig. 1. This merger standard accepts a higher cost increase for a given level of quality improvement than the TW standard and, in this way, can waive socially undesirable, costly mergers. The mechanisms described in Besanko and Spulber [4] and Lyons [5] no longer work; the simple CW standard cannot be preferable to the TW standard.

The probability of type II errors is, however, limited as long as hospitals show profit-maximizing behavior as in Fig. 1. When hospitals intrinsically value quality as in Fig. 2, they tend to accept more cost increase in return for a given level of quality improvement. As can be seen, this is the region where the simple CW standard commits type II errors. In other words, there is more chance that the simple CW standard approves socially undesirable mergers when hospitals are (partially) quality maximizing than in the case of profit-maximizing hospitals. The probability of committing a type I error, in contrast, decreases. Quality-maximizing hospitals initiate fewer mergers that lower quality. In this respect, quality-maximizing hospitals are more restrictive in proposing mergers than the standard of the competition authority and that is why there are less proposals that the standard rejects.

Regarding the extended CW standard in the case of partially-quality-maximizing hospitals (left panel of Fig. 2), no Type II error is committed. Because the probability of Type I error also decreases due to a hospitals’ behavior, decisions based on TW and extended CW welfare standard converge.

In conclusion, quality-maximizing behavior of hospitals has similar effects to the application of the simple CW standard, i.e., cost effects play less of a role in decisions. Scrutinizing whether cost increase is proportional to quality improvement is thus essential when hospitals attach high intrinsic value to quality. The simple CW standard is not concerned with the complete cost effects of a merger, therefore this standard may not be hard enough to block costly, quality-improving mergers. The use of extended CW is essential in this case because the external effects ignored by the simple CW are significant.

As shown, we can draw a parallel between a merger analysis based on extended CW (including the external effects) in hospital mergers and a merger analysis based on CW standard in non-health care mergers. Consequently, the advantages and disadvantages of the extended CW standard in the case of hospital mergers compared to the TW standard coincide with that of the CW standard compared to the TW standard in non-health care mergers since explicit consideration of externality is not needed in any of the cases. The results in the general literature on the application of CW or TW in merger analysis, summarized in the section on Related literature, apply to the considerations on extended CW standard versus TW standard in hospital mergers. We argue here for applying the extended CW or TW standard for health care markets, but avoiding the usage of the simple CW standard, which neglects significant external effects.

## Application in merger control

Most experiences with merger control in the hospital market have been in the US, Germany, and the Netherlands. In order to assess the practical value of our analysis, we review briefly how our findings can be related to current practice. We also help interpretation of our suggestions by a short hypothetical example.

It is important to note that there is some discrepancy between the approach of theoretical models to merger analysis and the practice of competition authorities. First, competition authorities make the trade-off between positive and negative effects required by the welfare standard analysis only when the expectation is that the merger harms competition on the relevant market significantly. The logic behind this is that a more intrusive or far-reaching merger control would require information that authorities typically do not have. Second, in many jurisdictions, again for understandable and pragmatic reasons, most merger decisions are made by and large on purely legalistic grounds. 15 For example, if market share is deemed low, a merger is waived without looking at the substantive side of a case, assuming implicitly that mergers between firms with relatively low market share are unlikely to cause problems. This implies that discussions on the choice of welfare standards are relevant only for those cases where a real welfare analysis is made.

In practice, the primary concern of competition authorities in hospital merger cases is the expected price change in the hospital market. Although other aspects of competition, such as quality, might also be considered, 16 evaluations of the effects of mergers are, in the first instance, based on the expected effects on the financial side of the market. Furthermore, effects are not geared towards consumers (i.e., premiums), but the analysis stops at the intermediate stage of insurer (i.e., hospital prices). As shown in this article, price effects at this intermediate level do not include externalities since the insurer has to bear the entire health care costs of enrollees. Using the notation of our model, the calculations address, in the first instance, the expenditure of insurers (∑_*i*=1_^*n*^ *D*_*i*_ *w*_*i*_) and not consumer welfare (PW − *S*∑_*i*=1_^*n*^ *D*_*i*_ *w*_*i*_). Consequently, the calculations of competition authorities are not distorted as long as the merger does not effect patient welfare.

Concerns may arise when the positive effects of a merger have to be weighed against negative price effects. Merging parties may want to show through an efficiency defense that benefits from quality improvements outweigh the welfare losses of possible price increases. Note that the burden of proof that quality improvements will emerge lies with the merging parties, but authorities have to trade these gains off against the competitive harm of the merger 17 An exact definition of ‘consumer’ then becomes essential in order to calculate the exact gains for consumers. When calculating the PW derived from improved quality, there are two essential questions to answer: who benefits from the quality improvement and what is their willingness to pay for it. Competition authorities should be aware that, through health insurance, a large group of patients is affected by the merger but their willingness to pay for potential quality improvements probably varies. By calculating the financial effects of a merger, the ‘consumer’ is defined as all enrollees since the price effect is calculated only at the insurers’ level, and is not extended to consumers. Considering the quality effects, many of these consumers may not benefit directly from the potential quality improvement and have, therefore, a low valuation of it.

The fact that clients of an insurer do not benefit equally from a given quality improvement of a provider can be captured in the divergence of their reported willingness to pay. Diener [30] distinguishes three sources of willingness to pay that can also be related to different consumer groups in insured population. 18 First, people value a good or service because of its ‘use value’. If they consume it directly, they are probably willing to pay a certain price for it. Second, people may expect that, in the future, they will probably need the good or service. In this case, they may be willing to pay an ‘option value’ for the possibility of access in the future. Finally, consumption of some goods and services has external effects. For example, a high uptake of vaccination in a population gives protection also to those not receiving the vaccine. People may be willing to pay for such ‘externality value’ of a good or service. Smith [31] shows that these three values differ. Although use value dominates, option value and externality value are also a significant source of the total value. Clients of an insurer are willing to pay either use value or the option value dependent whether they are current patients of the hospital. In a merger, a control process is therefore necessary to consider the heterogeneities in willingness to pay in order to come to an appropriate aggregation of patient benefits owing to the merger.

To illustrate this point, take the example of two hospitals that promise to invest in the latest technology in their dialysis center after they merge. We assume that this implies a real quality improvement in their dialysis treatment, which cannot be achieved without the merger due to scale efficiencies. The competition authority concludes from its research that the concentration is likely to considerably lessen competition on the hospital market. The merging parties, however, claim that this negative price effect would be compensated by the improved quality for consumers (the superior technology in the dialysis center). How should the authority trade off the price increase against the quality improvement? 19 Clearly, patients currently visiting the dialysis center are going to value the improvements (use value). Consumers on the given hospital market are also going to value it because might they need kidney dialysis, and can expect a better quality service in their hospital. Their valuation (option value) is however lower. Consumers on other hospital markets, but in the same insurance pool, do not benefit from the improvement; and therefore, have a very low (or zero) valuation. The competition authority has to take into account this diversity in willingness to pay when calculating the aggregated gains for consumers. It could be a pitfall to measure the willingness to pay of patients benefiting directly from the quality improvement, and to generalize it to all consumers on the hospital market. Increase in PW calculated in this way overestimates the real benefits to consumers. It should not be automatically assumed that all patients on the relevant hospital market have the same willingness to pay. Therefore, which consumer groups are included in the analysis, and how their welfare is aggregated, should be scrutinized.

The important conclusions of this article for legal practice are two-fold. First, potential gains for consumers have to be aggregated across the whole group covered by insurance. The reason is that the financial side and price effects are also calculated for this group because the price of hospital services rather than the insurance premium is considered in the analysis. Second, consumers probably attach diverging values to given improvements in health care according to their current situation and expected future needs, which has to be taken into account in the analysis.

## Conclusions

Our paper contributes to the literature in four ways. First, we have shown that consumer welfare in health care markets should be interpreted differently than in standard markets. This difference is prompted by the fact that mergers may have consequences for other consumers than just those directly affected. The indirect effect runs through health insurance premiums, i.e., if, after a merger, the bargaining position of the merged entity drives the premium up, all clients of the insurers are affected, not just patients of the merged hospital. This external effect on consumers paying premiums but actually not receiving health care has to be taken into account in the welfare standard of competition authorities.

Second, we have more specifically shown that failing to incorporate the above-mentioned external effect can make CW, in the case of quality enhancing mergers, a weaker standard than TW. This result contrasts with results in markets without significant externalities. The CW standard in such markets is always at least as tough as the TW standard. A further contrast to non-health care markets, i.e., a consequence of forgoing external effects, is that simple CW can commit both type I and type II errors in merger control. If the externality is positive (e.g., owing to a cost-reducing merger), TW enhancing mergers can be blocked, while if the externality is negative (e.g., owing to a cost increasing merger that raises premiums), TW reducing mergers that should be blocked can be waived. A CW standard that also includes external effects (i.e., the extended CW) corrects for these disadvantages, and is similar in its results to CW in non-health care markets.

Third, the non-profit nature of hospitals increases the probability that quality-improving mergers are proposed since hospitals put much weight on the intrinsic value of quality improvement. If these improvements go hand in hand with high costs, TW can be negatively affected. The more weight hospitals put on quality compared to monetary profit in their merger decisions, the more important it becomes to apply a merger standard that involves complete cost and quality effects. In conclusion, the simple CW standard is not suitable for health care markets; extended CW should be used instead.

Finally, the distinction between simple and extended CW in the practice of merger control can be reflected in the exact definition of the ‘consumer’ used in welfare analysis. The choice of definition is particularly important when a merger has both price and quality effects because the group of consumers affected directly by quality improvements (or deteriorations) usually does not correspond with the population bearing the financial consequences of the merger. The externality caused by insurance is reflected in the diverging valuation of quality improvements by different groups of consumers. The conclusions for merger control in health care markets is that it is essential to scrutinize which consumer groups are involved in the welfare analysis and how their benefits (or losses) from the merger are aggregated.

## Footnotes

1 The standpoint that competition law applies to health care just like to any other sector in the US stems from the case Goldfarb vs Virginia State Bar in 1975 [3].

2 There is no consensus in the literature about what (not-for-profit) hospitals maximize. See section on Objective function of hospitals for a overview of proposed alternatives.

3 The level of premium depends on the total consumption of health care services (at macro level). Increased consumption due to, for example, moral hazard problems causes premiums to rise. In this article, we do not consider the problem of moral hazard and abstract from possible links between consumption level and the premium. This is an acceptable assumption in health care systems such as those of the United Kingdom, the Netherlands, Denmark and Norway, where patients need a GP referral to visit a hospital.

4 The leading condition on the model of the hospital market that implies our main result is that clients of an insurer do not benefit equally from quality improvements in a specific hospital. This condition assumes a model in which not all consumers choose the same hospital even if that hospital is of higher quality. If there is diversity in hospital choice among consumers (e.g., due to horizontal differentiation of hospitals as in the circular city model), then our main result holds. Therefore, the circular city model is not restrictive.

5 We use the term patient to refer to a consumer who needs hospital treatment.

6 We do not assume a specific solution of this game but use the general efficiency condition of cooperative games: the negotiating hospital-insurer pair shares the total net gain from their cooperation (contract).

7 Any other proportion would be possible and would not alter the qualitative results of our model.

8 Note that PW_−i_ ≠ PW − PW_i_ (and TE_−i_ ≠ TE − TE_i_). The left hand side describes patient welfare (total expenditure) calculated for *n* − 1 hospitals in the market, while the right hand side represents patient welfare (total expenditure) in part of the market.

9 The hospital’s objective function (profit-, quality-maximization or partially both) matters in the merger decision of hospitals. See also sections on Objective function of hospitals and Merger decision of hospitals.

10 This is the consequence of our assumption on uniform insurance premiums. If premium differentiation among consumers is possible, then every patient (group) can bear its own financial burden; i.e., there would not be externality effect and hospital mergers would not differ from non-health care mergers. Co-payments are a tool of premium differentiation. However, co-payments generally do not shift the whole cost difference to patients but only part of it, so only affecting the quantitative nature of our results.

11 First, considering the extended definition of consumer, TW is the sum of CW and suppliers surplus, i.e., TW = (PW − ∑_*i*=1_^*n*^ *D*_*i*_ *w*_*i*_) + (∑_*i*=1_^*n*^ *D*_*i*_ *w*_*i*_ − ∑_*i*=1_^*n*^ *D*_*i*_ *c*_*i*_). Second, considering the restricted definition of consumer and so calculating with the simple CW, we can write TW as TW = (PW − *S* ∑_*i*=1_^*n*^ *D*_*i*_ *w*_*i*_) + (∑_*i*=1_^*n*^ *D*_*i*_ *w*_*i*_ − ∑_*i*=1_^*n*^ *D*_*i*_ *c*_*i*_) − ((1 − *S*) ∑_*i*=1_^*n*^ *D*_*i*_ *w*_*i*_). The first term equals the simple CW, the second term is the suppliers surplus, while the third term is the externality effect, i.e., effect on other consumer groups in the society. In the calculations of TW, externalities have to be taken into account since they form costs (or benefits) for society although not for the group defined as ‘consumers’ or ‘suppliers’ in the analysis. This can be compared to the textbook example of production or consumption externalities. The individual and social costs of consumption diverge, which results in externalities that are not considered by the consumers but that are costs for society. See, e.g., in [20, p 304].

12 The article closest to ours, Calem et al. [11], defines the goal of non-profit hospitals as outcome maximization. Quality maximization is an alternative hypothesis that is also supported by a number of empirical studies. Quality maximization is a form of perquisite maximization defined and tested in Chang and Jacobson [21]. Further, Malani et al. [26] could also not reject the hypothesis that hospitals maximize non-contractible quality.

13 The assumption that the change in quality is merger specific means that other hospitals cannot change their quality level. This is a realistic assumption when, for example, scale effects play a role.

14 As explained in the introduction, we find the non-evident definition of consumer the specialty of health care markets. Different definitions of the consumer yield different result in the welfare analysis. Therefore considering hospital mergers requires careful analysis.

15 Here we refer to the sequential approach in welfare analysis used, for example, in the EU, the UK and Australia. In the first step, general presumptions (e.g., on market shares) are applied to filter out unambiguous cases, i.e., when a positive or negative outcome of the analysis is highly probable. A thorough analysis is made only on the remaining, more ambiguous, cases. The US applies a case-by-case approach, i.e., every merger case is subject to a welfare analysis [1].

16 Quality as an attribute of competition is mentioned only in one-third of court cases in the US between 1985 and 1999 [28].

17 Efficiencies have to fulfill three criteria in order to be taken into account by the authorities: they have to benefit consumers, be merger specific and be verifiable [29]. Even if these requirements are fulfilled, there are some clear-cut cases when explicit trade-off of the price and quality effects are not necessary (e.g., a marginal quality improvement that increases the costs substantially). Weighing positive and negative effects against each other is the most relevant when both are significant and the net effect cannot be foreseen without a thorough analysis.

18 In his article, Diener discusses the application of contingent valuation methodology in health care. The contingent valuation method aims to elicit information on willingness to pay for, for example, a health care program or medical device. In this sense, the principals of eliciting the correct willingness to pay in the population raises the same question as applying the appropriate price-quality trade-off in merger control.

19 This is a hypothetical example. To the best of our knowledge, quality and price effects have not yet been set explicitly against each other by competition authorities.
